# Bacteria Display Differential Growth and Adhesion Characteristics on Human Hair Shafts

**DOI:** 10.3389/fmicb.2018.02145

**Published:** 2018-09-07

**Authors:** Swat Kim Kerk, Hui Ying Lai, Siu Kwan Sze, Kee Woei Ng, Artur Schmidtchen, Sunil S. Adav

**Affiliations:** ^1^Lee Kong Chian School of Medicine, Nanyang Technological University, Singapore, Singapore; ^2^School of Materials Science and Engineering, Nanyang Technological University, Singapore, Singapore; ^3^Nanyang Environment & Water Research Institute, Interdisciplinary Graduate School, Nanyang Technological University, Singapore, Singapore; ^4^School of Biological Sciences, Nanyang Technological University, Singapore, Singapore; ^5^Skin Research Institute of Singapore, Singapore, Singapore; ^6^Wound Healing Centre, Bispebjerg University Hospital, Copenhagen, Denmark; ^7^Division of Dermatology and Venereology, Department of Clinical Sciences, Lund University, Lund, Sweden

**Keywords:** hair shaft, *S. aureus*, S. epidermidis, P. aeruginosa, E. coli

## Abstract

Apart from the skin surface, hair represents a significant tissue component with a capacity of bacterial interactions. New information can be obtained about hair function through the characterization of bacterial adherence, colonization, and responses to hair shafts *per se.* In this proof-of-principle study, we examine the growth kinetics of Gram-positive *Staphylococcus aureus* and *Staphylococcus epidermidis,* and Gram-negative *Pseudomonas aeruginosa* and *Escherichia coli* in the presence of human hair shafts. We explore the ability of these bacteria to adhere to and colonize hair shaft surfaces, as well as the resulting impact on the hair’s surface morphology. We show that hair shafts inhibit the growth of Gram-positive *S. aureus* and *S. epidermidis*, while the growth kinetics of *P. aeruginosa* and *E. coli* remain unaffected. Scanning electron microscope analysis and steeping studies show that *P. aeruginosa* and *E. coli* to adhere to and colonize on human hair shafts without significantly affecting the hair shaft’s surface morphology. *P. aeruginosa* produced a substantial amount of biofilm on the hair shaft surfaces, while *E. coli* specifically inhabited the edges of the cuticle scales. Taken together, our results demonstrate differences in bacterial responses to human hair shafts, which may provide novel insights into hair and scalp health.

## Introduction

The human hair shaft is keratinized fibrous tissue that grows from follicles beyond the surface of the epidermis. Hair plays a key role in body temperature regulation, defense, protection from the environment, and aesthetics, as well as acting as a sensory organ. Human skin nurtures an estimated one million bacteria per square centimeter of skin (Grice et al., 2008), and the scalp houses miscellaneous common scalp commensal microflora (Clavaud et al., 2013; Sommer et al., 2015; Wang et al., 2015). Scalp disorders including folliculitis types, fungal diseases, dandruff, and folliculitis decalvans, among others, are caused by or linked to microbes, which play a key role in disease predisposition and pathogenesis (DeAngelis et al., 2005; Grimalt, 2007; Wang et al., 2015).

Molecular and biochemical methods have shown that the most abundant species present on dandruff scalps are *Malassezia restricta* and *Malassezia globosa*(Gemmer et al., 2002; Clavaud et al., 2013). Other studies have focused on the balance between *Staphylococcus*, *Propionibacterium,* and *Malassezia,* emphasizing that dysfunctional balance between them leads to scalp disorders (Clavaud et al., 2013; Park et al., 2017). Balance of the bacterial population is thus fundamentally connected to limiting fungal growth, scalp health, and disorders, but the interaction of these bacterial community members with the hair shaft has not been elucidated. From the viewpoint of host defense mechanisms, it is important to study the ability of bacteria to adhere and replicate and their interaction with resident and commensal microflora.

Members of the genus *Staphylococcus* are common colonizers of the skin, including *Staphylococcus aureus* and *Staphylococcus epidermidis* (Otto, 2009). *Staphylococcus* predominantly colonizes the hands, chest, abdomen, nose, and axillae scalp (Otto, 2010). There is a tendency of these skin areas to stay moist, which promotes adherence and colonization of bacteria. In contrast, the surfaces of hair shafts remain dry and hydrophobic. The skin flora travels toward the upper hair follicle, where it easily is dislodged and transferred to surfaces upon touching (Jarvis, 1994), where it persists for a prolonged time (Brooke et al., 2009).

Furthermore, hair shafts are constantly exposed to the environment and can be a potential site for harboring bacteria due to its grooved cuticle surface and long, thin structure. However, hair has not been evaluated for bacterial adherence and colonization. In hospital operating theaters and industrial clean areas, surgeons and associated staff are required to cover their scalp hair to avoid the spread of bacteria and prevent microbial infections. However, there is no experimental evidence demonstrating the adherence of bacteria and their colonization on the hair shaft. Better understanding of the bacterial ecology of the hair shaft and hair–microbe interactions may provide novel insights into the spread and growth of bacteria on hair.

Bacterial profiling of hair was demonstrated to be a useful forensic tool to augment existing forensic techniques (Tridico et al., 2014). The bacterial community structure of the human hair shaft has been profiled by thermal gradient gel electrophoresis (TGGE), which revealed approximately 20 bacterial species including *Pseudomonas* sp. (Yun et al., 2010). A metagenomics analysis of bacteria on human scalp hair revealed high diversity with 4,838 core bacteria and 1,220 transient bacteria (Tridico et al., 2014). The bacterial community of hair consists of Gram-positive and Gram-negative bacteria. *S. aureus* and *S. epidermidis* have been identified on human skin and scalp (Grice and Segre, 2011). *Escherichia coli* was isolated from hair in 72% of out-patients, 61% of in-patients, and 46% of medical and nursing staff (Summers et al., 1965). Bacteria including *Klebsiella pneumoniae*, *S. pyogenes*, *Pseudomonas aeruginosa*, *Streptococcus pneumoniae*, *Serratia marcescens,* and *E. coli* were isolated and linked with alopecia areata (Morales-Sanchez et al., 2010). *P. aeruginosa* can cause cutaneous infections (Chiller et al., 2001) Hence, hypothetically, apart from *staphylococci*, bacteria such as *P. aeruginosa* and *E. coli* can be spread via human hair, and potentially cause infections. The study of bacterial interaction with human hair may therefore be of conceptual importance and highlight possible new roles of hair shafts in the complex skin- bacteria interaction, of relevance for skin and wound infection, or scalp disorders. Therefore, using a reductionistic approach we decided to study *S. aureus* and *S. epidermidis*, and *P. aeruginosa* and *E. coli,* as representative members of Gram-positive and Gram-negative bacteria, respectively.

We focused on evaluation of bacterial behavior in the presence of a hair shafts in order to detect possible differential interactions of the respective bacteria with human hair, and to delineate the mechanism of adherence and colonization on the surface of the hair surface. The impact of bacterial colonization on the surface morphology of the hair shaft was also studied using scanning electron microscopy (SEM).

## Materials and Methods

### Hair Samples, Bacterial Strains, Culture Conditions, and Growth Curve

Hair samples derived from the distal parts (usually representing 10–30% of the total hair length, away from the scalp) were obtained from healthy donors with written consent. The study protocol was approved by Nanyang Technological University Institutional Review Board (IRB-2016-11-042). The donors had no dandruff, scalp diseases, or chemical treatments such as hair dyes, bleach, or perms. After collection, the hair shafts underwent two cycles of disinfection with 70% ethanol, followed by washing three times with MilliQ water for 2 min in a vortex (**Supplementary Figure S1**, right panel). This process is referred to as “standard washing.”

Bacterial strains *S. epidermidis* (ATCC^®^ 14990), *S. aureus* (ATCC^®^ 29213), *E. coli* (ATCC^®^ 25922), and *P. aeruginosa* (ATCC^®^ BAA-47^TM^) were procured from the American Type Culture Collection (ATCC) and maintained according to the supplier’s protocol. The strains were initially grown in LB broth medium (1^st^ Base, Singapore) or TSB medium (Merck, Branchburg, NJ, United States). *E. coli*, *P. aeruginosa*, *S. aureus* were cultured in LB broth while *S. epidermidis* in TSB at 37°C with constant shaking at 150 rpm. The bacterial cultures (1 ml) were harvested at an optical density (OD) of about 0.7 to 0.8, washed with sterilized MilliQ water and re-suspended in sterilized MilliQ water (1 ml). Hundred μl of bacterial suspension was added to 100 ml sterilized M9 minimal medium constituting 12.8 g l^−1^ of Na_2_HPO_4_⋅7H_2_O, 3.0 g l^−1^ of KH_2_PO_4,_ 0.5 of NaCl g l^−1^, 1.0 g l^−1^ of NH_4_Cl, 0.19 g l^−1^ of amino acid mix, 2.0 g l^−1^ of C_6_H_12_O_6_ (glucose), 1.0 g l^−1^ of yeast extract, and the following micronutrients: 0.300 g l^−1^ of MgSO_4_⋅7H_2_O, 0.005 g l^−1^ of FeSO_4_⋅7H_2_O, 0.075 g l^−1^ of MnSO_4_⋅H_2_O, 0.03 g l^−1^ of CaCl_2_⋅2H_2_O, 0.002 of CoCl_2_, 0.1 g l^−1^ of thiamin, and bacterial strains were re-cultured.

The bacterial cultures (OD ∼0.5 in M9 minimal medium) were thereafter diluted 10-fold in M9 minimal medium. If required, OD was further adjusted to 0.05 using fresh sterilized M9 minimal media. Two hundred mg of hair shafts were added to this OD adjusted bacterial culture (10 ml) and the mixture was incubated at 37°C with shaking at 150 rpm, and OD at 600 nm was recorded every hour using a spectrophotometer (BioTek Instruments, Inc.). Growth curves of bacteria without hair and with hair alone in the same media were used as a control. Hair samples used for this study were about 3–9 cm away from the scalp. After collection, hair samples were prepared by adopting two cycles of disinfection with 70% ethanol, followed by washing three times with MilliQ water for 2 min during vortexing. Then, the hairs samples were dried at 50°C. The hair length used for this experiment was about 3–5 cm.

For the solution killing assay, the bacteria *E. coli, P. aeruginosa,* and *S. aureus* were cultured in LB broth while *S. epidermidis* in TSB medium overnight at 37°C with constant shaking at 150 rpm. The overnight grown bacterial cultures were sub-cultured in 4 ml of freshly sterilized LB or TSB medium until mid-log phase (OD ∼0.5–0.6). The mid-log phase culture was adjusted to OD 0.05 and 200 mg of hair shafts were added to 10 ml of these cultures, and incubated at 37°C at 150 rpm for 3 h. Hundred μl of the suspension at early mid-log phase (OD = 0.2), mid-log phase (OD = 0.5) and stationary phase (OD = 0.8 or above) were 10-fold serially diluted and 3 μl of each dilution was spotted on sterile LB agar plates. The plates were maintained at room temperature for 15 min, incubated at 37°C overnight and then images were acquired using ChemiDoc imaging system (Bio-Rad Laboratories, Inc., United States).

### Analytical Methods and Scanning Electron Microscopy (SEM)

Seven donor’s hair shaft samples were tested for bacterial growth behavior with a constant quantity of hair shafts. The media, their composition, and experimental procedure were the same as above. A steeping study was performed with 20-mg samples of hair shafts. The hair shafts were steeped in the bacterial culture broth having OD of 0.05 for 3 h at 37°C, collected, washed three times with sterile PBS buffer in a vortex, and kept gently on LB agar. The LB agar plates were incubated at 37°C overnight, and pictures were taken using a camera. To analyze the effects of soluble factors from the hair shafts, the hair shaft wash solution was collected at each stage (**Supplementary Figures S1**, **S2**, right panel) and tested for their effects on the growth of both Gram-positive and Gram-negative bacteria. In brief, hair shafts (0.2 g) were disinfected with 70% ethanol (2 min with vortexing), followed by further three washing steps with MilliQ water for (2 min with vortexing). This hair shaft preparation procedure was considered as standard wash (SW). The washing solutions after each MilliQ wash were collected and labeled, 1^st^ SW, 2^nd^ SW, 3^rd^ SW, and 4^th^ SW as shown in flow chart in **Supplementary Figure S1** (right panel). Similarly, hair shafts were subjected to extensive wash (EW). The test procedure and wash solution sampling points are shown in **Supplementary Figure S2** (flow chart, right panel). Hundred μl of wash solution collected at each step was added to 100 μl bacterial cultures in 96-well plate. The plate was incubated at 37°C with constant shaking and bacterial growth was monitored using OD measurement.

In another experiment, hair shafts were suspended in M9 minimal media (containing 0.1% yeast extract and 0.2% glucose) and autoclaved at 121°C for 15 min. The media was cooled, equilibrated at 37°C, and inoculated with test bacteria. The bacterial growth was then monitored at 600 nm. Experiments were performed five times, and the mean data with SD are presented.

To test the efficiency of shampoo, 500 μl of mid-log phase bacteria (OD_600__nm_ = 0.05) was mixed with 5 ml of 1% sterile agarose solution, and the mixture was poured evenly on top of a sterile 1.5% nutrient base agar in a Petri dish. Upon solidification, a sterile paper disk was placed on top, and 10 μl of 0.01, 0.1, or 1% shampoo was pipetted on the paper disk. The shampoo preparations were diluted with MilliQ water. The plates were incubated for 10 min at room temperature and then at 37°C overnight. The inhibition zone was recorded using a ChemiDoc Imaging System (Bio-Rad Laboratories, Inc.).

A residual concentration of shampoo was not detected by a foaming test, and we determined the minimum inhibitory concentration of shampoo. To mimic the routine use of shampoo, we washed hair shafts (200 mg) with 0.01% concentrations of different brand shampoos, rinsed them with MilliQ water twice, and dried them using a dryer. The hair shafts were suspended in M9 media containing 0.1% yeast extract, inoculated with a bacterial culture and adjusted to 0.05 OD at 600 nm. The mixture was incubated at 37°C with constant shaking (150 rpm), and OD at 600 nm was recorded at 5 h using a spectrophotometer (BioTek Instruments, Inc.).

Next, we explored the mechanism of bacterial adherence to the human hair shafts and studied the impact of bacterial adherence on the shafts’ surface morphology. To this end, *S. epidermidis*, *S. aureus*, *E. coli,* and *P. aeruginosa* were inoculated into M9 minimal media, adjusted to OD 0.05 and then 0.2 g of hair shafts were added, followed by incubation at 37°C with constant shaking at 200 rpm. The samples were harvested at different time intervals ranging from 5 to 120 h (OD ∼0.08 to 1.2). The hair shafts were collected, washed three times with sterile MilliQ water, and suspended in 1x PBS buffer. The hair shaft samples were then fixed with 4% paraformaldehyde in 0.05 M PBS for 8 h and dehydrated for 20 min each in increasing concentrations of ethanol (30, 40, 50, 70, 90, and 100%). The dehydrated hair shafts were coated with gold, and the hair structure, surface morphology, and bacterial adherence were observed using a SEM (Jeol JSM-5310, Tokyo, Japan). About 4–5 hair strands at two-three locations per hair were imaged for each bacterial species per experiment. SEM imaging were repeated at least three-times in separate independent experiments.

### Statistical Analysis

Unless stated otherwise, all experiments were performed independently at least five times, and data are presented as the mean values ± standard deviation (SD). The statistical significance of differences between experimental groups was determined through one-way ANOVA or two-way ANOVA. Differences were considered significant at *P* < 0.05.

## Results

### Effect of Hair Shafts on Bacterial Growth

**FIGURE 1:**
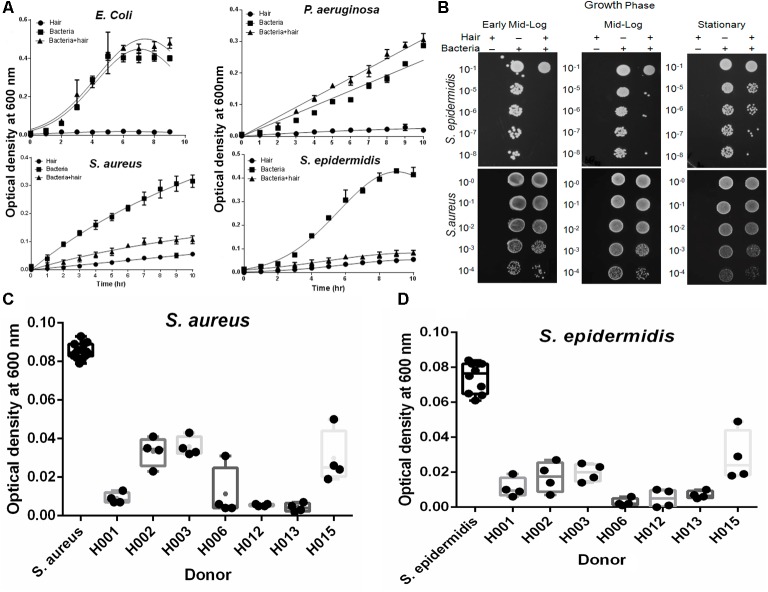
Effect of hair on the growth kinetics of bacteria. **(A)** The growth kinetics of bacteria is plotted as the optical density profile at 600 nm as a function of time. The lines show Gaussian curve fit using GraphPad Prism. Hair shafts from donor H006 were used for this study. The results represent the means (± SD) of five independent experiments. Statistical significance of differences is analyzed by one-way ANOVA. **(B)** Solution killing assay at early, mid-log and stationary phase to examine bacterial growth inhibition by human hair shafts. Hair shafts from donor H006 were used for this study. **(C)** Growth inhibition of *Staphylococcus aureus* was studied in different donor hair samples at 5 h and the recorded OD is shown (box plots). Statistical significance is analyzed by one-way ANOVA. **(D)** Growth inhibition of *Staphylococcus epidermidis* was studied in the different donor hair samples. Statistical significance is analyzed by one-way ANOVA.

The behavior of skin-colonizing bacteria on human hair shafts was studied. Gram-positive *S. aureus* and *S. epidermidis* cause nosocomial infections, and Gram-negative *E. coli* and *P. aeruginosa* are common infectious agents. Under standard growth conditions in the absence of hair, both Gram-positive and Gram-negative bacteria follow well-known growth kinetics with a short lag phase of 1 to 2 h (**Figure 1A**). In contrast, the presence of hair shafts significantly (*P* < 0.0001) inhibited the growth of *S. epidermidis* and *S. aureus.* However, the growth of Gram-negative *E. coli* and *P. aeruginosa* was not affected by the shafts. The solution killing assay revealed growth inhibition of *S. epidermidis* and *S. aureus* in the early-log, mid-log, and stationary phases (**Figure 1B**). The extension of this study with more donor samples (*n* = 7) yielded comparable results of the growth inhibition of *S. aureus* and *S. epidermidis* in the early mid-log phase (**Figures 1C,D**).

### Exploration of Antibacterial Effects

**FIGURE 2:**
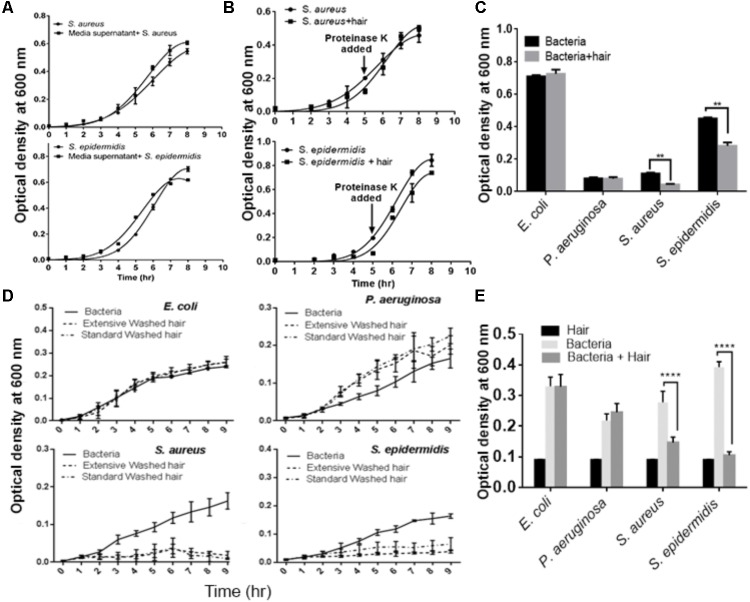
Evaluation of the effects of extrinsic factors on the growth of microbes cultured with hair shafts. **(A)** Evaluation of effects of media inhibition. **(B)** Growth profile of bacterial species in presence of proteinase K. **(C)** Analysis of growth behavior in presence and absence of hair shaft under static condition. **(D)** Evaluation of the impact of standard and extensively washed hair shafts on the growth of bacterial species. **(E)** Analysis of autoclaved hair shafts for their effects on the growth of bacterial species. Statistical significance is analyzed by one-way ANOVA. Statistical significance of differences is analyzed by two-way ANOVA, ^∗∗^*P* ≤ 0.01, ^∗∗∗∗^*P* ≤ 0.0001.

We performed a series of experiments to determine the factors that influence bacterial growth. Wash solutions collected at each stage of a standard wash were tested for their effects on the growth of the bacterial species. The results showed no effects on bacterial growth (**Supplementary Figure S1**), indicating that the growth of *S. aureus* and *S. epidermidis* was inhibited by the physical presence of the hair shafts. To confirm this further, we incubated hair shafts with growth media for 10 h with constant shaking (200 rpm), collected supernatant and then, this supernatant was inoculated with *S. aureus* and *S. epidermidis.* The growth profile showed no statistically significant inhibition when compared with the control (**Figure 2A**). However, bacterial growth inhibition by hair shafts in media (**Figure 1A**) was found to be abolished by added proteinase K treatment (**Figure 2B**). To evaluate whether growth inhibition of these strains is a function of shaking of the hair samples, we studied growth profile in static conditions. The results revealed significant inhibition of the growth of *Staphylococcus* species (**Figure 2C**). An extensive washing of hair shafts (**Supplementary Figure S2**, right panel) and the testing of wash solutions at each stage revealed that the bacterial inhibition was not due to extrinsic factors (**Supplementary Figure S2**). Notably, the standard and extensively washed hair shafts again significantly inhibited the growth of *S. aureus* and *S. epidermidis* (**Figure 2D**).

We next tested for the possibility that the hair shafts used could be contaminated with unknown bacteria that may inhibit the growth of the test bacteria. The unwashed, disinfected, and washed hair shafts were incubated in LB agar medium at 37°C for 72 h. However, the results revealed no bacterial colonies (**Supplementary Figure S3**). In another experiment, we autoclaved the hair shaft samples and studied their impact on microbial growth. The autoclaved shafts continued to inhibit the growth of *S. aureus* and *S. epidermidis,* but not *E. coli* and *P. aeruginosa* (**Figure 2E**). Thus, these series of experiments revealed that intrinsic factors in the hair shafts *per se* cause the observed growth inhibition of *S. aureus* and *S. epidermidis.*

**FIGURE 3:**
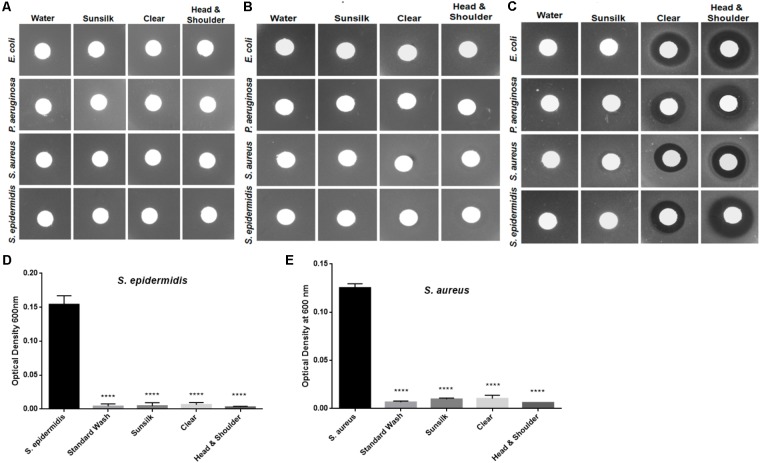
Analyses of possible residual antibacterial activity at different shampoo concentrations: **(A)** 0.01%, **(B)** 0.1%, and **(C)** 1.0%. **(D)** Hair shafts were washed with shampoo and tested for *S. epidermidis* growth behavior after 5 h incubation. Statistical significance of differences is analyzed by one-way ANOVA, ^∗∗∗∗^*P* ≤ 0.0001. **(E)** Hair shafts were washed with shampoo and tested for *S. aureus* growth behavior after 5 h incubation. Statistical significance is analyzed by one-way ANOVA, ^∗∗∗∗^*P* ≤ 0.0001.

The hair shafts were further tested for residual concentrations of shampoos using a foaming ability test, which revealed no residual shampoo on the hair shafts. The hair shaft donors indicated that they used distinct shampoo brands in routine hair washing (**Supplementary Table S1**). We evaluated the potency of these shampoos at concentrations of 0.01 to 1% (**Figures 3A–C**), which revealed that the minimum inhibitory concentration of these shampoos is more than 1%. In other words, ≥1% shampoo is required to inhibit the growth of bacteria. The residual concentration of shampoo after regular hair washes was estimated not to exceed 0.01–0.1%. With this reasoning, as well as the fact that the shafts were extensively washed before experiments, it is unlikely that there were any possible confounding effects on bacteria induced by residual shampoos.

Furthermore, to mimic shampoo use and hair washing, we washed the hair shaft samples with the shampoos, cleaned them with water, and tested the hair shafts for bacterial growth behaviors. The growth of *S. aureus* and *S. epidermidis* was again inhibited, in contrast to *E. coli* and *P. aeruginosa* (**Figures 3D,E**). Taken together, the observations in these experiments revealed that bacterial growth inhibition was affected by the physical presence of the hair shafts.

### Bacterial Adherence to Human Hair Shafts

**FIGURE 4:**
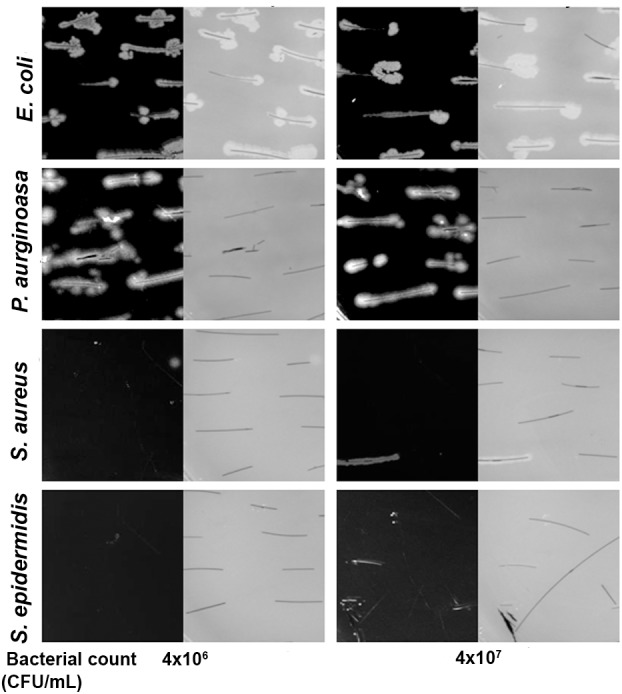
Bacterial adherence to the hair shaft by plate culturing technique. Both black and gray images at different CFU are shown since it is difficult to visualize the hair shafts against a black background. The hair shafts were steeped in the bacterial culture broth (initial OD 0.01 and 0.05) for 3 h at 37°C. The hair shafts were collected, washed three times with sterile PBS buffer, and placed gently on LB agar. The LB agar plates were thereafter incubated at 37°C overnight, and pictures were taken using a digital camera.

**FIGURE 5:**
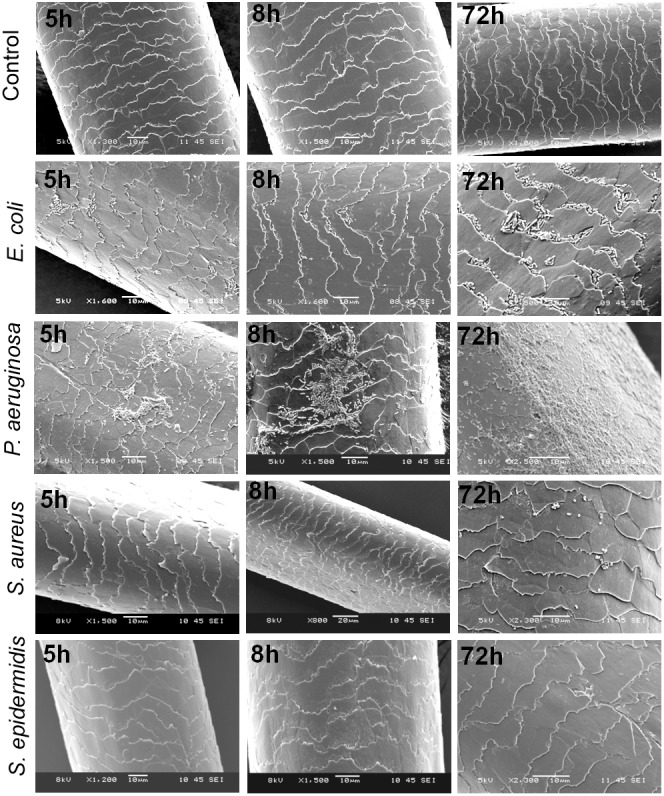
Scanning electron micrographs (SEMs) of hair shafts incubated with bacteria to examine their adherence at different time intervals from 5 to 72 h. Hair shafts were incubated separately with *S. epidermidis*, *S. aureus*, *Escherichia coli,* and *Pseudomonas aeruginosa* (initial OD∼0.05) at 37°C with constant shaking at 200 rpm. The hair shaft samples were harvested at different time intervals ranging from 5 to 120 h (OD ∼0.08 to 1.2), washed with 1x PBS and fixed and processed for SEM (see section “Materials and Methods”).

The steeping test indicated that *E. coli* and *P. aeruginosa* adhere to the hair shafts, while *S. epidermidis* and *S. aureus* showed very few or no colonies (**Figure 4**). To confirm the bacterial adherence to the shafts, we performed an SEM analysis and found a sizeable number of colonies of *E. coli* and *P. aeruginosa* on the surface of the hair shafts at all tested time points (**Figure 5**). Interestingly, *P. aeruginosa* formed a biofilm on the hair shaft surface, and colonies were noted all over the surface. In contrast, *E. coli* was observed only along the edges of the cuticle scales, presumably due to the physical sheltering that those locations provide against fluid flow. Thus, the mechanism of adherence and colonization of these bacteria on hair shafts differs significantly.

**FIGURE 6:**
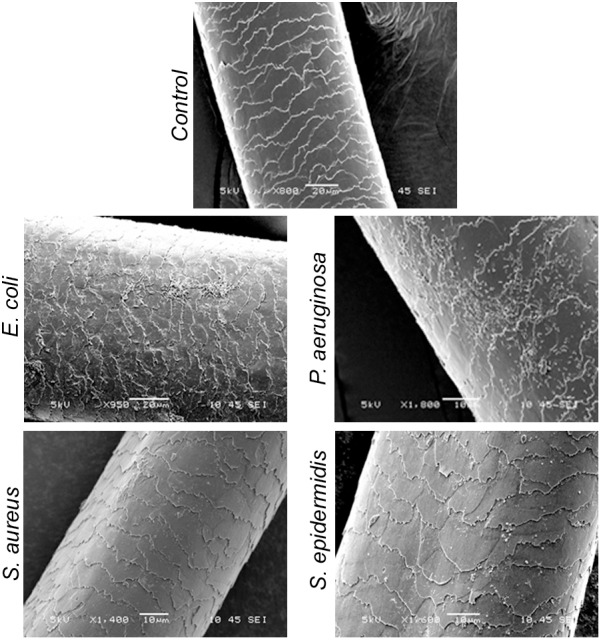
Scanning electron micrographs of hair shafts incubated with bacteria to examine their adherence. Hair shafts were incubated in bacterial broth of *S. epidermidis*, *S. aureus*, *E. coli,* and *P. aeruginosa* (OD ∼0.2) for 3 h. The hair shaft samples were collected, washed with 1x PBS buffer, fixed with 4% paraformaldehyde, dehydrated, and imaged using SEM.

As shown in the SEM images, *S. epidermidis* and *S. aureus* were not visible in the early phase at 5 h. Upon prolonged incubation for 72 or 120 h, only a few colonies were noted on the surface of the hair shafts. Further, we incubated hair shafts in the bacterial broth (OD_600__nm_ 0.21) for 3 h with constant shaking at 200 rpm, washed them thrice with 1x PBS, fixed as stated in the method section and imaged using SEM. The results revealed adherence of *E. coli* and *P. aeruginosa* (**Figure 6**). In other words, S. *epidermidis,* *S. aureus, E. coli,* and *P. aeruginosa* display different interactions with the hair shafts. The surface morphology of the hair shafts was not altered due to the colonization of the bacteria.

## Discussion

We investigated the interaction and growth kinetics of Gram-positive *S. aureus* and *S. epidermidis* and Gram-negative *E. coli* and *P. aeruginosa* in the presence of human hair shafts. For the first time, we showed the colonization and adherence of *E. coli* and *P. aeruginosa* on hair shafts, where *P. aeruginosa* formed a biofilm, while *E. coli* inhabited only the edges of the cuticle scales. Furthermore, this study demonstrates significant antibacterial effects of human hair shafts on *S. aureus* and *S. epidermidis*.

Hair-derived antimicrobial proteins or peptides have indeed been identified in hair shafts (Adav et al., 2018; Subbaiah et al., 2018). Interestingly, histones, which are well-known antimicrobial agents (Patat et al., 2004), were detected abundantly in the hair shaft proteome (Adav et al., 2018; Subbaiah et al., 2018). The fact that the experiments showed no evidence of the release of soluble antimicrobials from the hair shafts in the conditions used, raises the possibility that hair surface itself acts as an antimicrobial scaffold, where non-soluble antimicrobials mediate antibacterial effects. For example, antimicrobial histones (Subbaiah et al., 2018) could alter the cationicity and hydrophobicity of the hair shaft surface. In this context, this would in fact resemble the function of synthetic biomaterials having antimicrobial (non-releasable) coatings or antibacterial surfaces (Hasan et al., 2013; Iolanda et al., 2017). Clearly, further studies, well-beyond the scope of this work are required to identify the exact components of the hair shaft surfaces which selectively inhibit the growth of these *staphylococci*, and the mode(s) of action of these substances. The steeping study and SEM observations revealed that *P. aeruginosa* and *E. coli* adhere to and colonize hair shaft surfaces without damaging them. A prerequisite for bacterial colonization is adherence to the host tissue through specific adhesin-receptor mechanisms. The physicochemical characteristics of bacterial surface architecture like surface charge and hydrophobicity (Cree et al., 1994) also play a role in the mechanisms of bacterial adherence to the hair shaft. Although no firm adherence of ATCC strains of *S. epidermidis* and *S. aureus* was noted in this study, the clinical isolates of methicillin-resistant *S. aureus* (MRSA) and methicillin-resistant *S. epidermidis* (MRSE) were found to colonize on the hair shaft (Mase et al., 2000). However, when 32 Swedish and 27 German MRSA isolates were tested for their adherence to host tissues, inconsistent adhesion was noted, which may be due to the genetic variability among them (Cree et al., 1994). The members of *staphylococci* adhere to host tissue through surface proteins such as fibronectin, fibrinogen, vitronectin, laminin, and von Willebrand factors, but with varying degree of specificity (Otto, 2010). The hair shaft proteome identified fibrinogen but in a very low abundance (Adav et al., 2018). Thus, a relative lack of this protein could be another possible cause of non-adherence of these bacteria. Difference in bacterial cell wall components among Gram-positive and Gram-negative strains or differences in their responses to the hair shafts may also be other reasons for the observed differential growth and adhesion characteristics.

From a clinical perspective (Xu et al., 2016) investigated the intra- and inter-associations of scalp dandruff and found decreased levels of *Propionibacterium* and increased *Staphylococcus* density in regions of dandruff. Similarly, Clavaud et al. (2013) noted the reciprocal inhibition of scalp bacteria *Propionibacterium* and *Staphylococcus.* It is well-known that *Propionibacterium* secretes bacteriocins to suppress the growth of *Staphylococcus* (Scharschmidt and Fischbach, 2013), while *Staphylococcus* ferments glycerol, which is naturally produced in the skin to inhibit the overgrowth of *Propionibacterium* (Wang et al., 2014). Although *Propionibacterium* and *Staphylococcus* are highly abundant on the human scalp, our study found growth inhibition by hair shafts for only *S. aureus* and *S. epidermidis*. Consistent with our observations, Wakeam et al. (2014) also noted that clean-shaven workers were significantly colonized with *S. aureus* and meticillin-resistant coagulase-negative *Staphylococci* (MRSA) compared to unshaved males. In other words, the growth of *S. aureus* and MRSA was inhibited by facial hair. Also of interest is that shaving a body part prior to surgery results in increased risk for surgical site infections (Lefebvre et al., 2015), an observation compatible with the results from our study.

Recently, hair shafts have become significant biological specimens in the fields of forensic toxicology and clinical chemistry as alternatives to blood and urine samples for drug abuse screening purposes (Boumba et al., 2006). Although hair shafts are the most ubiquitous physical evidence (Opel et al., 2008; Nilsson et al., 2012), DNA analysis using hair samples is difficult due to limited sample amounts and DNA stability. However, metagenomics analysis of the hair shaft microbiome may provide alternative evidence to augment other forensic results (Tridico et al., 2014). For example, metagenomics analysis revealed the *Lactobacillus* taxon on pubic hair, which helped to determine gender, as these taxa are highly dominant in female pubic hair (Tridico et al., 2014). Further detailed research is required to identify possible personalized traits in the human hair shaft microbiome.

## Conclusion

This study has demonstrated the unique response of Gram-positive *S. aureus* and *S. epidermidis* and Gram-negative *E. coli* and *P. aeruginosa* to human hair shafts. A series of experiments indicated that the growth inhibition of *S. aureus* and *S. epidermidis* was due to hair shaft components. Future studies are clearly needed to identify antimicrobials and other hair surface factors that selectively inhibit *S. aureus* and *S. epidermidis*, as well as to understand their exact mode and mechanism of inhibition. Furthermore, we showed the adherence and colonization of *E. coli* and *P. aeruginosa* on hair shaft surfaces, with *P. aeruginosa* being observed all over the hair shaft surface while *E. coli* colonized only along the edges of cuticle scales, suggesting a different mechanism of adherence.

## Author Contributions

AS, SA, and SK were involved in the experimental design. SK and SA performed the experiments. HL contributed to the SEM experiments. SA, KN, and AS actively contributed to the writing of the manuscript. AS, KN, and SS provided resources and materials.

## Conflict of Interest Statement

The authors declare that the research was conducted in the absence of any commercial or financial relationships that could be construed as a potential conflict of interest.
