# Supplementary material for: Bacteria Display Differential Growth and Adhesion Characteristics on Human Hair Shafts
Source: Front Microbiol. 2018 Sep 7;9:2145. doi: 10.3389/fmicb.2018.02145 (PMC6137140; doi:10.3389/fmicb.2018.02145)
Supplement: Supplementary file 1 [file Data_Sheet_1.pdf]

Bacteria display differential growth and adhesion characteristics on human hair shafts

Swat Kim Kerk[1], Hui Ying Lai[3,4], Siu Kwan Sze[2], Kee Woei Ng[3,4,5], Artur Schmidtchen[1,6,7],

Sunil S Adav[1,2]*

[1]Lee Kong Chian School of Medicine, Nanyang Technological University, 59 Nanyang Drive, Singapore, 636921, [2]School of Biological Sciences, Nanyang Technological University, 60 Nanyang Drive, Singapore 637551, [3]School of Materials Science and Engineering, Nanyang Technological University, Singapore, [4]Nanyang Environment & Water Research Institute, Interdisciplinary Graduate School, Nanyang Technological University, Singapore, [5]Skin Research Institute of Singapore, Singapore, [6]Wound Healing Centre, Bispebjerg University Hospital, Copenhagen, Denmark, [7]Division of Dermatology and Venereology, Department of Clinical Sciences, Lund University, Lund, Sweden

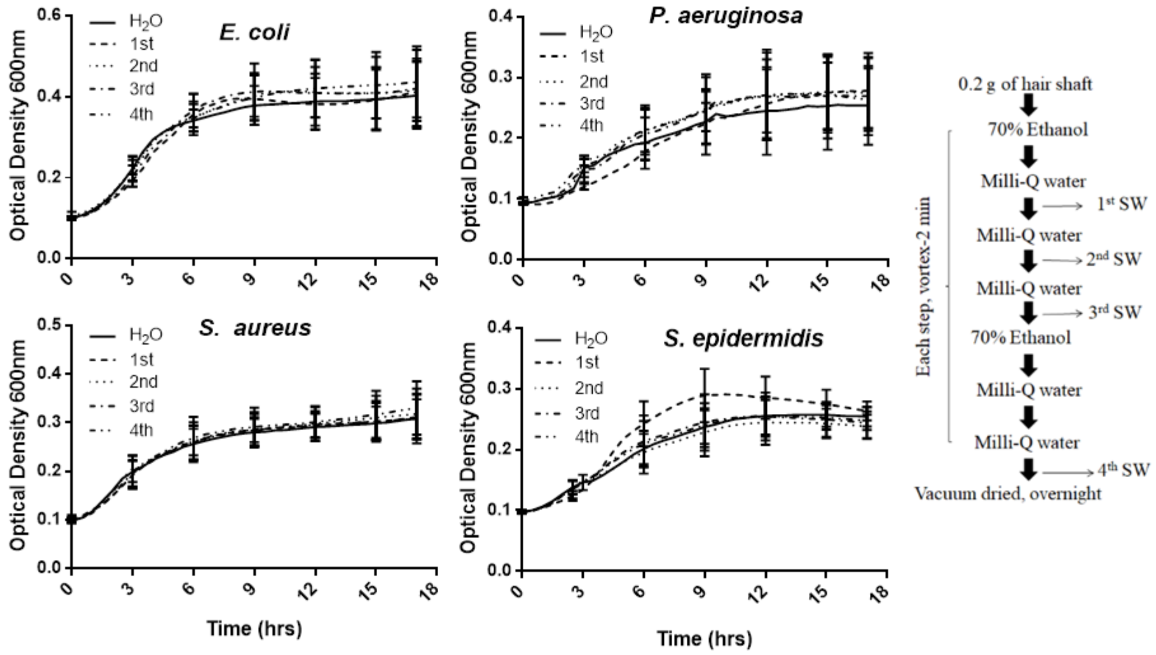

Figure S1. Evaluation of standard wash solution. Hair shafts were prepared for the experiment by adopting the washing as shown in the right panel, washings were collected and labeled as 1st-4th SW (Standard Wash) and analyzed for antimicrobial activity. Statistical significance is analyzed by two-way ANOVA.

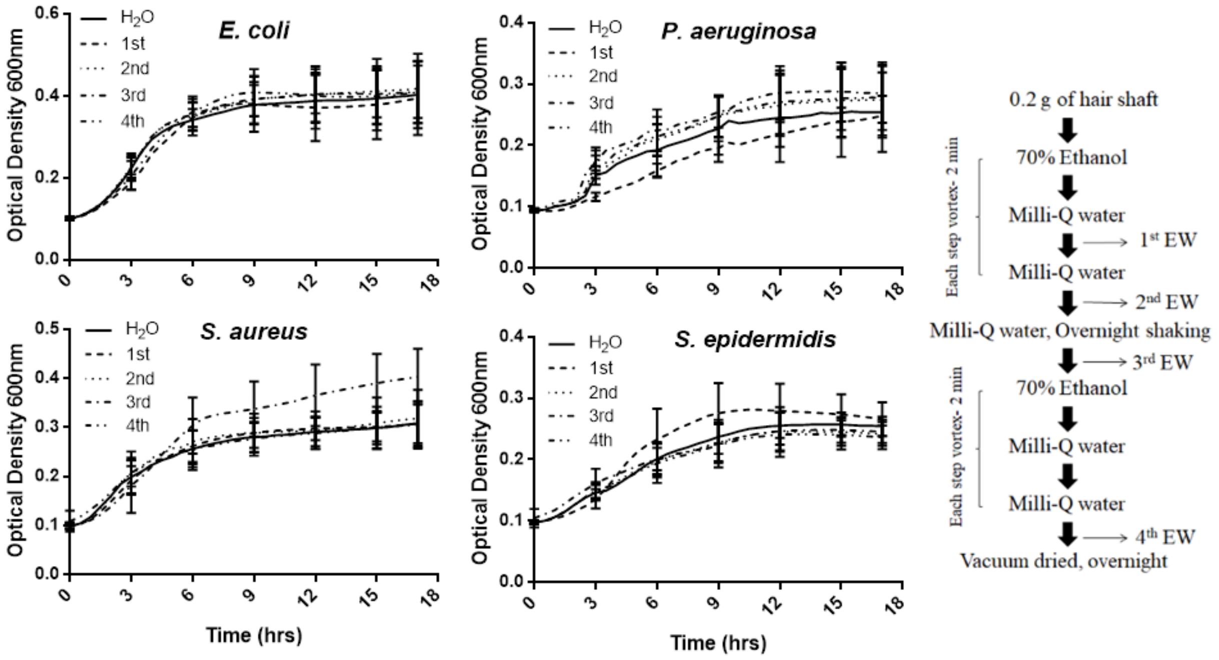

Figure S2. Evaluation of extensive wash solution for antimicrobial activity. Hair shafts were washed extensively by adopting the washing as shown in the right panel, washings were collected and labeled as 1st-4th EW (extensive wash) and analyzed for antimicrobial activity. Statistical significance is analyzed by two-way ANOVA.

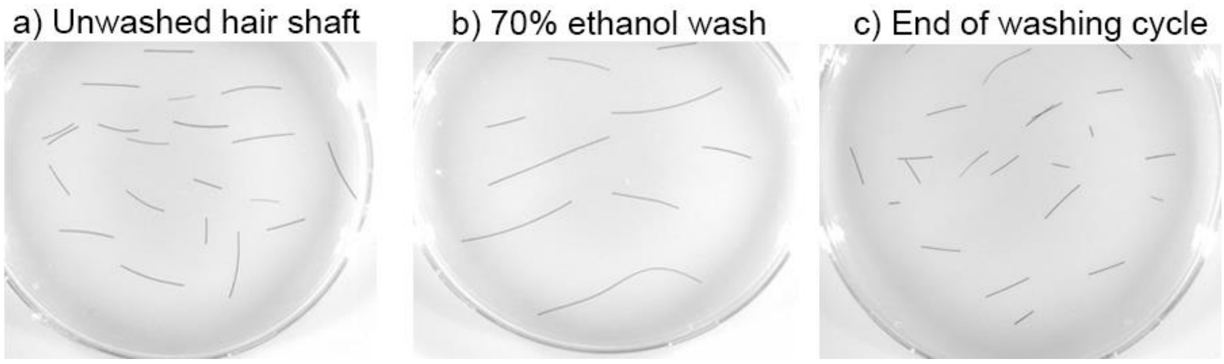

Figure S3. Evaluation of hair shafts for bacterial contamination. The hair shaft samples were incubated with LB agar in a petri dish 72 h at 37 °C and evaluated for bacterial contamination a) The hairs shafts sample before subjected to washing, b) disinfected with 70% ethanol, c) complete standard wash hair shafts.

Table S1. Hair shaft donor’s details. The donor’s donated hair shaft at morning was after a bath and that donated hair at evening has taken bath at morning.

Donor ID	Gender	Age	Shampoo Brand	Hair Wax	Chemical Treatment	Hair donation time
H001	Male	28	Clear	Yes	No	Morning
H002	Male	25	Sunsilk	Yes	No	Evening
H003	Male	24	Sunsilk	No	No	Evening
H006	Male	38	Loreal	No	No	Evening
H012	Female	25	Head & Shoulders	No	No	Morning
H013	Male	18	Head & Shoulders	No	No	Morning
H015	Male	28	Dove	No	No	Evening
